# Alternative initiations with 6-monthly paliperidone palmitate. A descriptive study

**DOI:** 10.1192/j.eurpsy.2023.2295

**Published:** 2023-07-19

**Authors:** O. C. Sobrino, B. Sergio

**Affiliations:** 1 Rey Juan Carlos Hospital, Madrid; 2 Hospital Infanta Elena, Madrid, Spain

## Abstract

**Introduction:**

6-monthly paliperidone palmitate features an initiation regimen through 1-monthly paliperidone palmitate or 3-monthly paliperidone palmitate. Some patients don´t have sufficient adherence to treatment and it is necessary at the clinical level to start directly with 6-monthly paliperidone palmitte. There is une little clinical experience with these alternative initiations and throuht this work those that have been carried out for 12 months at the Rey Juan Carlos Hospital are exposed.

**Objectives:**

The main objective of the study is to describe the alternative initations performed with 6-monthly paliperidone palmitate in routine clinical practice, having opted for a regimen different from the standard for clinical reasons.

**Methods:**

A retrospective selection of patiens will be made throuht non-probabilistic consecutive sampling, including all patients who have benn administered 6-monthly paliperidone palmitate with a stard different form the standard during the last 6 months. To do his, the electronic medical record will be used, first selecting the patients who have started 6-monthly paliperidone palmitate through the anonymized digital records and, later, including in the study only those who have followed and alternative initiation pattern. The variables studied will be the following: age, sex, diagnosis, dose of paliperidone palmitate, initiation regimen, consumption of toxic substances, absenteeism from 6-monthly paliperidone palmitate, visits to the emergency room and admissions.

**Results:**

The study included a total of 5 patients ( n:5). 80% of the patients were male and 20%were female. The mean age was 39.7 years. 80% of the patients had an associated substance use disorder. The following alternate sarting schedules were with biannual paliperidone palmitate: monthly paliperidone palmitate 150 mg together with semi-annual paliperidone palmitate both on day 1 ( n: 2) or monthly paliperidone palmitate 150 mg on day 1, monthly paliperidone palmitate 100 mg on day 5 and semi-annual paliperidone palmitate on day 12 ( n: 3 ).

A total of 0 visits to the emergency department and 0 admissions were observed after the 6-monthly paliperidone palmitate regimen.

**Conclusions:**

Alternative initations with 6-monthly paliperidone palmitate may be a useful and safe clinical alternative in patients with very low adherence who, due to clinical needs, require startin 6-monthly paliperidone palmitate earlier in order to guarantee adherence .

**Disclosure of Interest:**

None Declared

